# Prevalence and associated complications of supernumerary teeth in a clinical CBCT sample from the hail region, Saudi Arabia: a retrospective study

**DOI:** 10.3389/froh.2026.1817816

**Published:** 2026-05-07

**Authors:** Abdulrahman K. Alshammari, Muteb A. Algharbi, Freah L. Alshammary, Nabeel S. Almotairy, Hatem D. Alshammari, Amal R. Alrashidi, Ebtisam A. Alshdokhy, Alanoud S. Alshammari, Ahmed A. Madfa

**Affiliations:** 1 Department of Preventive Dentistry, College of Dentistry, University of Ha’il, Ha’il, Saudi Arabia; 2 Department of Orthodontics and Pediatric Dentistry, College of Dentistry, Qassim University, Buraidah, Saudi Arabia; 3 Dentist, College of Dentistry, University of Ha’il, Ha’il, Saudi Arabia; 4 Oral & Maxillofacial Surgery Resident, Ministry of Health, Ha'il, Saudi Arabia; 5 Department of Restorative Dental Science, College of Dentistry, University of Ha’il, Ha’il, Saudi Arabia

**Keywords:** CBCT, cone beam computed tomography, hyperdontia, orthodontic, Saudi Arabia, supernumerary teeth

## Abstract

**Objective:**

Cone beam computed tomography (CBCT) from dental records prior to orthodontic treatment was used in this investigation to determine the prevalence, characteristics, and associated consequences of Supernumerary teeth (ST).

**Methods:**

A retrospective analysis of 266 CBCT scans from patients ages 9 to 55 that were obtained between 2010 and 2025 was conducted. Every ST was evaluated for its forms, morphology, position, eruption status, closeness to neighboring roots, and associated complication. The chi-square or Fisher's exact tests and multivariate logistic regression were used. At α = 0.05, the threshold for statistical significance was established.

**Results:**

163 ST were present in 97 patients (36.6%). Among the most prevalent were premolars (38.8%), lateral incisors (21.4%), mesiodens, and canines (15.3% each). The mandible (19.0%) and left side (20.5%) had slightly higher ST rates than the maxilla (16.2%). The majority had impaction (73.5%), inclined position (37.8%), and supplement morphology (51.0%). The overall prevalence did not differ by sex ( *p* = 0.1885), but females showed more crowding and resorption ( *p* < 0.001), while males showed more impactions of ST.

**Conclusion:**

This CBCT study reported a high prevalence (36.6%) of ST in a Saudi subpopulation. The mandibular and premolar ST were the most frequently observed and the gender and arch difference was observed with significance. Impaction and delayed eruption were the most common complications. Due to the inherent issues with CBCT-based datasets, and the retrospective design may result in selection bias.

## Introduction

Teeth that grow in addition to the normal dentition and exceed the typical complement of the dental formula are known as Supernumerary teeth (ST), or hyperdontia (1). This anomaly can affect one or both dental arches, and manifest in multiple forms, unilaterally or bilaterally (2, 3). The front maxilla is the most common location for ST, followed by the lower arch premolar region (4, 5). ST may occur in both the permanent and primary dentitions, though they are relatively less prevalent in the primary dentition (2). These dental abnormalities can interfere with proper tooth eruption, impair occlusion, and have a detrimental effect on oral function and appearance, so early and accurate diagnosis is crucial (6).

ST can occur in both primary and permanent dentitions, with lower frequencies reported in the primary dentition (0.07%–0.6%) (1, 7–14). The prevalence of ST in the general population ranges from 2.4% to 6%. Male-to-female ratios range from 1.18:1 to 4.5:1, indicating a higher prevalence in men than in women (3–5, 15, 16). Additionally, some syndromes, such as cleidocranial dysplasia and Gardner's syndrome, are linked to ST (1, 7). The reported prevalence varies by population; southern Chinese (2.4%), Japanese (3.4%), African Americans (6%), and South Africans (7%) have higher rates than Caucasians (0.4%–2.1%) (17, 18). These differences are most likely due to differences in ethnicity, diagnostic methodologies, imaging modalities, and sample strategies (7).The reported prevalence of ST varies greatly by area in Saudi Arabia, ranging from 0.3% to 15.6% (12–14, 19–25). The majority of research indicates that there is no discernible gender distribution pattern and that they are more prevalent in the maxillary anterior region, particularly mesiodens (14, 19, 22, 24). Studies in the central region have found prevalence rates between 1.8% and 3.5% (19, 24). In the western region, the rates are lower (0.3%), and in the southern region, they are variable (0.5%–3.5%) (12, 14, 20, 21, 23). These differences are probably due to different diagnostic criteria, sampling methods, and population characteristics (8, 9, 16, 26, 27). There is no data from the northern regions, such as Hail. Furthermore, traditional 2D imaging techniques, including panoramic and periapical radiographs, possess intrinsic limitations in precisely localizing and characterizing ST due to anatomical overlap (2, 4). Cone-beam computed tomography, on the other hand, provides improved 3D visualization, which increases diagnostic precision and permits careful treatment planning, particularly in affected impacted teeth (15, 19).

Studies conducted in Saudi Arabia have not yet used CBCT imaging to evaluate the prevalence distribution and associated ST problems. This gap in the literature emphasizes the necessity for a more thorough and precise study using CBCT imaging techniques. Through the use of CBCT imaging, the study aimed to ascertain the occurrence, distribution, and morphological features of ST in the Saudi sub-population as well as to pinpoint related complications.

## Methods

### Study design

This retrospective observational study was conducted using cone-beam computed tomography (CBCT) records obtained from dental archives of patients treated between 2010 and 2025 at the Hail Dental Center, the College of Dentistry clinics, and a private dental practice in Hail City, Saudi Arabia. CBCT scans were originally acquired for orthodontic assessment. Ethical approval was obtained from the Research Ethics Committee of the University of Hail (Approval No. H-2023–349). The requirement for informed consent was waived due to the retrospective nature of the study. All data were anonymized prior to analysis.

### Sample selection

A consecutive sampling approach was employed, whereby all CBCT scans meeting the eligibility criteria within the study period were screened for inclusion. To ensure that the dataset represented unique individuals, only one CBCT scan per patient was included; when multiple scans were available, the earliest diagnostically adequate scan was selected. Referral bias was recognized as a possibility because CBCT imaging was carried out for clinical indications rather than research.

CBCT scans were included if they met the following criteria:

(1) patients aged ≥9 years at the time of imaging.
(2) CBCT images of sufficient diagnostic quality.

Exclusion criteria were:

(1) poor-quality CBCT images.
(2) History of prior orthodontic treatment.
(3) presence of craniofacial syndromes.
(4) Evidence of pathological lesions affecting dental structures.
(5) History of permanent tooth extraction.

Only scans fulfilling all eligibility criteria were included in the final analysis.

### Sample size

The minimum sample size was calculated using Cochran's formula {*N* = [Zα2 × P (1-P)]/D2} (28). where P represents the estimated prevalence of dental anomalies (80%, based on prior regional data (24), D is the desired precision, and Z is the standard normal deviate at a 95% confidence level (1.96). The calculated minimum sample size was 245 participants. To improve statistical reliability, a final number of 266 patients were enrolled.

### Outcome assessment

For all CBCT scans, a dentoalveolar field of view protocol was used. With specialized imaging software (i-Dixel, Morita Corp., Kyoto, Japan), the obtained data were reconstructed into successive slices and carefully assessed in three orthogonal planes: sagittal, coronal, and axial. The software's calibrated ruler and magnification tool were utilized when needed to ensure measurement accuracy and enhance viewing. A comprehensive examination comprising both qualitative and quantitative assessments was conducted on each discovered ST. The approach employed by Mossaz et al. (1) was the source for variable definitions, measurement techniques, and classification standards.

The morphology was initially categorized as conical, tuberculate, supplementary, odontoma-like, or a developing tooth bud (2). Then, eruption status was used to differentiate between impacted and erupted teeth ST, and the site in relation to normal eruption tooth was noted as normal, inclined, transverse, inverted, or indeterminate. We recorded the cusp tip of ST in relation to the long axis of the closest erupted adjacent tooth as apical to the root apex, within the apical, middle, or cervical third, or coronal to the cemento–enamel junction and catogrized the ST as (mesiodens, supplemental lateral incisor, canine, premolar, paramolar, or distomolar). Buccolingual location was identified as either palatal/lingual, intra-arch (median), or labial/buccal. Using standardized criteria, the degree of root resorption in teeth next to ST was evaluated and categorized as either no resorption, cervical third, middle third, or apical third involvement based on its vertical extent in relation to the root. Based on dentine involvement, the degree of resorption was further classified as none, slight (less than half of dentine thickness), moderate (more than half of dentine thickness without pulp exposure), or severe (related to pulp exposure). Any ST-related local disturbance affecting neighboring teeth, such as crowding (lack of space within the arch), spacing (excess interdental space), malposition (deviation from normal alignment), diastema (midline or interproximal space), impaction (failure of eruption within the expected time), delayed eruption (eruption later than the normal developmental period), and cyst formation (radiolucent lesion associated with ST), were operationally defined and documented as associated complications. These defined definitions were utilized to ensure the evaluations were consistent and reproducible.

Three experienced evaluators used monitors with standard brightness and resolution settings in optimal lighting conditions to independently assess the CBCT images. To ensure consistent interpretation, all examiners attended a calibration training session prior to the assessment. Under the direction of top professionals with more than 10 years of experience, including MAA, FLA, NSA, AAM, HDA, and examiner AKA, ARA, EAA, and ASA completed the calibration and assessment procedures. Predetermined criteria and acceptable deviations were rigorously followed in all evaluations prior to the start of the experimental phase. Three calibrated examiners independently evaluated all CBCT images under standardized viewing conditions. A calibration exercise was conducted prior to data collection. Inter-observer reliability was assessed using 20 randomly selected scans, yielding a kappa coefficient of 0.92, indicating excellent agreement. Blinded evaluation of the same scans was used to examine intra-observer reliability two weeks later, and the results showed great reproducibility. Discrepancies were resolved through consensus.

Data were analyzed using SPSS software (version 22; IBM Corp., Armonk, NY, USA). Analyses were made using descriptive statistics, such as percentages and frequencies. When appropriate, chi-square or Fisher's exact tests were used to evaluate associations between categorical variables. Multivariate regression analysis was performed to evaluate the independent association between gender and the presence of ST. Statistical significance was set at *p* < 0.05.

## Results

**Figure 1:**
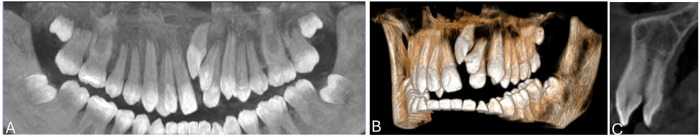
An illustration of a cone beam computed tomography (CBCT) scan showing two types of supernumerary teeth. A conical -shaped supernumerary tooth between tooth 13 & 12 and a tuberculate supernumerary tooth between tooth 21 & 22. (**A:** panoramic view; **B:** 3D, **C:** Sagittal view).

**Figure 2:**
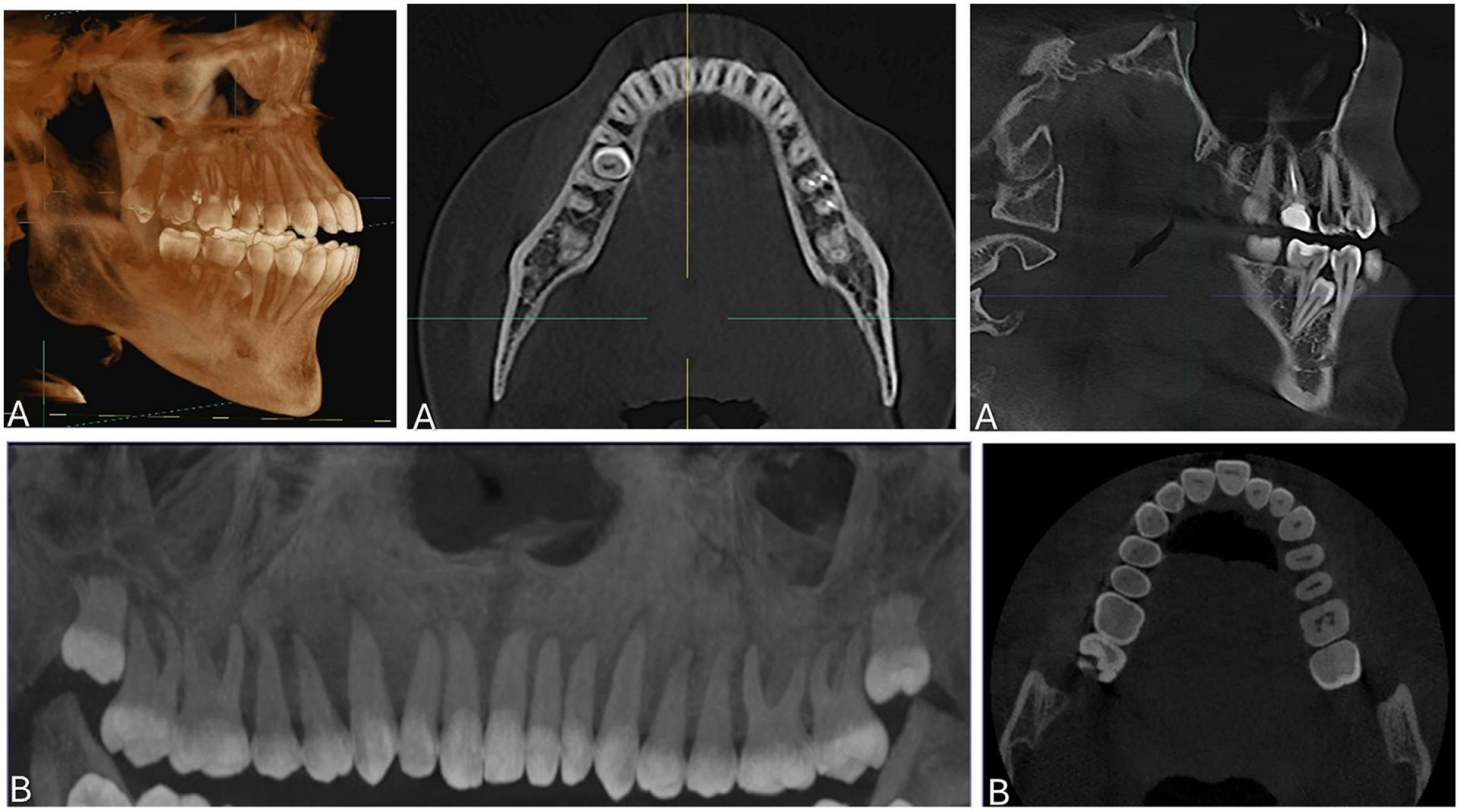
An illustration of a cone beam computed tomography (CBCT) scan showing example of a supplement-shaped of supernumerary teeth. **(A)** In the posterior mandibular lingual region. **(B)** In the maxillary anterior left side.

As shown in Table 1, the study included 266 patients, with a slight female predominance (54.1%). ST were identified in 36.6% of individuals. ST were slightly more frequent in the mandible (19.0%) than in the maxilla (16.2%), and marginally more common on the left side (20.5%) than the right (16.0%). In terms of type, premolars were the most common ST (38.8%), followed by lateral incisors (21.4%), while other types were less frequent. Morphologically, ST were predominantly supplemental in shape (51.0%) and mainly located on the palatal/lingual side (51.0%). Most ST were impacted (73.5%) and presented in inclined (37.8%) or normal (34.7%) orientations. Vertically, the cusp tip was most frequently located in the cervical third of the adjacent tooth root, as illustrated in Figure 1. Variations in tooth morphology are further demonstrated in Figure 2. Collectively, these findings indicate that ST in this population tend to be unerupted and positioned close to adjacent roots, which may have clinical implications for adjacent structures.

**Table 1:** Demographic and clinical characteristics of the study population.

Variable	Category	Frequency (*n*, %)
Gender	Male	123 (45.9%)
	Female	145 (54.1%)
Arch	Maxilla	47 (16.2%)
	Mandible	51 (19.0%)
Side	Right	43 (16.0%)
	Left	55 (20.5%)
Presence of supernumerary tooth	Yes	98 (36.6%)
	No	170 (63.4%)
Type of supernumerary tooth	Mesiodens	15 (15.3%)
	Lateral incisors	21 (21.4%)
	Canine	15 (15.3%)
	Premolars	38 (38.8%)
	Paramolars	3 (3.1%)
	Distomolars	6 (6.1%)
Vertical location of cusp tip	Apical to root tip	13 (13.3%)
	Apical third	15 (15.3%)
	Middle third	19 (19.4%)
	Cervical third	30 (30.6%)
	Coronal	21 (21.4%)
Buccolingual location of crown	Labial/Buccal	8 (8.2%)
	Midline/Within arch	38 (38.8%)
	Palatal/Lingual	50 (51.0%)
Shape of supernumerary tooth	Conical	20 (20.4%)
	Tuberculate	16 (16.3%)
	Supplemental	50 (51.0%)
	Odontoma	4 (4.1%)
	Developing tooth bud	8 (8.2%)
Position of supernumerary tooth	Normal	34 (34.7%)
	Inclined	37 (37.8%)
	Transverse	16 (16.3%)
	Inverted	3 (3.1%)
	Undefinable	8 (8.2%)
State of eruption	Erupted	26 (26.5%)
	Impacted	72 (73.5%)

The distribution of ST by gender (Table 2) showed no statistically significant difference (*p* = 0.185). This was further supported by logistic regression analysis (Table 3), which demonstrated that gender was not a significant predictor of ST occurrence (OR = 1.385, 95% CI: 0.468–1.269, *p* = 0.306). These results suggest that sex does not play a major role in the overall prevalence of ST.

**Table 2:** Distribution of supernumerary teeth according to gender.

Variables		Dental Anomalies		
		Yes	No	*p*-value
Gender	Male	49 (18.3%)	74 (27.6%)	.185
	Female	49 (18.3%)	96 (35.8%)	

**Table 3:** Binary logistic regression analysis predicting supernumerary teeth according to gender.

Predictor	B	Exp (B)	Wald	SE	df	*p*-value	95% CI for Exp(B)
Gender	−.260	1.385	1.046	.254	1	.306	.771 (.468–1.269)

Despite the lack of association with prevalence, several characteristics of ST varied significantly by gender (Table 4). Significant differences were observed in ST type (*p* < 0.001), vertical cusp tip location (*p* = 0.001), tooth shape (*p* = 0.001), root resorption location (*p* = 0.022), and associated complications (*p* < 0.001). In contrast, buccolingual position of crown of ST (*p* = 0.135), overall tooth position (*p* = 0.057), eruption status (*p* = 0.069), and degree of root resorption (*p* = 0.085) were not significantly different. Although these findings indicate some gender-related variation in ST characteristics, the lack of consistent patterns across variables limits their epidemiological significance.

**Table 4:** Relationship between gender, type of supernumerary tooth and associated complications with supernumerary teeth.

Variable	Gender	Category	Frequency (*n*, %)	*p*-value
Type of supernumerary tooth	Male	Mesiodens	11 (11.2%)	<0.001
		Lateral incisors	14 (14.3%)	
		Canines	12 (12.2%)	
		Premolars	10 (10.2%)	
		Paramolars	2 (2%)	
		Distomolars	0 (0%)	
	Female	Mesiodens	4 (4.1%)	
		Lateral incisors	7 (7.1%)	
		Canines	3 (3.1%)	
		Premolars	28 (28.6%)	
		Paramolars	1 (1%)	
		Distomolars	6 (6.1%)	
Vertical location of cusp tip	Male	Apical to root tip	1 (1%)	0.001
		Apical third	5 (5.1%)	
		Middle third	9 (9.2%)	
		Cervical third	24 (24.5%)	
		Coronal	10 (10.2%)	
	Female	Apical to root tip	12 (12.2%)	
		Apical third	10 (10.2%)	
		Middle third	10 (10.2%)	
		Cervical third	6 (6.1%)	
		Coronal	11 (11.2%)	
Bucco-lingual location of crown	Male	Labial/Buccal	4 (4.1%)	0.135
		Median/within arch	15 (15.3%)	
		Palatal/Lingual	28 (28.6%)	
	Female	Labial/Buccal	4 (4.1%)	
		Median/within arch	23 (23.5%)	
		Palatal/Lingual	22 (22.4%)	
Shape of supernumerary tooth	Male	Conical	13 (13.3%)	0.001
		Tuberculate	15 (15.3%)	
		Supplemental	19 (19.4%)	
		Odontoma	0 (0%)	
		Developing tooth bud	2 (2%)	
	Female	Conical	7 (7.1%)	
		Tuberculate	1 (1%)	
		Supplemental	31 (31.6%)	
		Odontoma	4 (4.1%)	
		Developing tooth bud	6 (6.1%)	
Position of supernumerary tooth	Male	Normal	24 (24.5%)	0.057
		Inclined	14 (14.3%)	
		Transverse	7 (7.1%)	
		Inverted	0 (0%)	
		Undefinable	4 (4.1%)	
	Female	Normal	10 (10.2%)	
		Inclined	23 (23.5%)	
		Transverse	9 (9.2%)	
		Inverted	3 (3.1%)	
		Undefinable	4 (4.1%)	
State of eruption	Male	Erupted	9 (9.2%)	0.069
		Impacted	40 (40.8%)	
	Female	Erupted	17 (17.3%)	
		Impacted	32 (32.7%)	
Degree of root resorption	Male	No resorption	46 (46.9%)	0.085
		Slight	0 (0%)	
		Moderate	3 (3.1%)	
		Severe	0 (0%)	
	Female	No resorption	40 (40.8%)	
		Slight	3 (3.1%)	
		Moderate	3 (3.1%)	
		Severe	3 (3.1%)	
Root resorption of adjacent teeth	Male	No resorption	46 (46.9%)	0.022
		Cervical	0 (0%)	
		Middle	3 (3.1%)	
		Apical	0 (0%)	
	Female	No resorption	38 (38.8%)	
		Cervical	2 (2%)	
		Middle	5 (5.1%)	
		Apical	4 (4.1%)	
Associated complications	Male	Asymptomatic	6 (6.1%)	<0.001
		Spacing	3 (3.1%)	
		Crowding	3 (3.1%)	
		Malposition	1 (1%)	
		Diastema	0 (0%)	
		Impaction	17 (17.3%)	
		Delayed eruption	17 (17.3%)	
		Cyst	1 (1%)	
		Other	1 (1%)	
	Female	Asymptomatic	6 (6.1%)	
		Spacing	10 (10.2%)	
		Crowding	9 (9.2%)	
		Malposition	13 (13.3%)	
		Diastema	1 (1%)	
		Impaction	5 (5.1%)	
		Delayed eruption	5 (5.1%)	
		Cyst	0 (0%)	
		Other	0 (0%)	

Side-based comparisons (Table 5) revealed significant differences in ST type (*p* < 0.001), tooth shape (*p* = 0.012), position (*p* = 0.004), eruption status (*p* = 0.042), and associated complications (*p* < 0.001). However, no significant differences were found for vertical cusp tip location (*p* = 0.435), buccolingual position of crown of ST (*p* = 0.751), root resorption degree (*p* = 0.195), or location (*p* = 0.723). Overall, these results suggest that while some lateral variations exist, they are not consistent enough to indicate a strong side-related pattern.

**Table 5:** Relationship between side, type of supernumerary tooth and associated complications with supernumerary teeth.

Variable	Side	Category	Frequency (*n*, %)	*p*-value
Type of supernumerary tooth	Right	Mesiodens	15 (15.3%)	<0.001
		Lateral incisors	13 (13.3%)	
		Canines	6 (6.1%)	
		Premolars	10 (10.2%)	
		Paramolars	3 (3.1%)	
		Distomolars	0 (0%)	
	Left	Mesiodens	0 (0%)	
		Lateral incisors	8 (8.2%)	
		Canines	9 (9.2%)	
		Premolars	28 (28.6%)	
		Paramolars	0 (0%)	
		Distomolars	6 (6.1%)	
Vertical location of cusp tip	Right	Apical to root tip	3 (3.1%)	0.435
		Apical third	6 (6.1%)	
		Middle third	10 (10.2%)	
		Cervical third	24 (24.5%)	
		Coronal	4 (4.1%)	
	Left	Apical to root tip	10 (10.2%)	
		Apical third	9 (9.2%)	
		Middle third	9 (9.2%)	
		Cervical third	6 (6.1%)	
		Coronal	17 (17.3%)	
Bucco-lingual location of crown	Right	Labial/Buccal	4 (4.1%)	0.751
		Median/within arch	20 (20.4%)	
		Palatal/Lingual	21 (21.4%)	
	Left	Labial/Buccal	4 (4.1%)	
		Median/within arch	18 (18.4%)	
		Palatal/Lingual	29 (29.6%)	
Shape of supernumerary tooth	Right	Conical	11 (11.2%)	0.012
		Tuberculate	13 (13.3%)	
		Supplemental	19 (19.4%)	
		Odontoma	4 (4.1%)	
		Developing tooth bud	0 (0%)	
	Left	Conical	9 (9.2%)	
		Tuberculate	3 (3.1%)	
		Supplemental	31 (31.6%)	
		Odontoma	0 (0%)	
		Developing tooth bud	8 (8.2%)	
Position of supernumerary tooth	Right	Normal	28 (28.6%)	0.004
		Inclined	10 (10.2%)	
		Transverse	5 (5.1%)	
		Inverted	0 (0%)	
		Undefinable	4 (4.1%)	
	Left	Normal	6 (6.1%)	
		Inclined	27 (27.6%)	
		Transverse	11 (11.2%)	
		Inverted	3 (3.1%)	
		Undefinable	4 (4.1%)	
State of eruption	Right	Erupted	8 (8.2%)	0.042
		Impacted	39 (39.8%)	
	Left	Erupted	18 (18.4%)	
		Impacted	33 (33.7%)	
Degree of root resorption	Right	No resorption	42 (42.9%)	0.195
		Slight	3 (3.1%)	
		Moderate	2 (2%)	
		Severe	0 (0%)	
	Left	No resorption	44 (44.9%)	
		Slight	0 (0%)	
		Moderate	4 (4.1%)	
		Severe	3 (3.1%)	
Root resorption of adjacent teeth	Right	No resorption	40 (40.8%)	0.723
		Cervical	2 (2%)	
		Middle	4 (4.1%)	
		Apical	1 (1%)	
	Left	No resorption	44 (44.9%)	
		Cervical	0 (0%)	
		Middle	4 (4.1%)	
		Apical	3 (3.1%)	
Associated complications	Right	Asymptomatic	2 (2%)	<0.001
		Spacing	3 (3.1%)	
		Crowding	1 (1%)	
		Malposition	2 (2%)	
		Diastema	1 (1%)	
		Impaction	18 (18.4%)	
		Delayed eruption	19 (19.4%)	
		Cyst	0 (0%)	
		Other	1 (1%)	
	Left	Asymptomatic	10 (10.2%)	
		Spacing	10 (10.2%)	
		Crowding	11 (11.2%)	
		Malposition	12 (12.2%)	
		Diastema	0 (0%)	
		Impaction	4 (4.1%)	
		Delayed eruption	3 (3.1%)	
		Cyst	1 (1%)	
		Other	0 (0%)	

Comparisons between the maxilla and mandible (Table 6) showed largely similar distributions for most variables. No significant differences were observed in ST type (*p* = 0.537), vertical position (*p* = 0.327), tooth shape (*p* = 0.607), eruption status (*p* = 0.099), root resorption degree (*p* = 0.465), root resorption location (*p* = 0.184), or associated complications (*p* = 0.138). Significant differences were noted only in buccolingual position of crown of ST (*p* = 0.031) and overall tooth orientation (*p* = 0.001). However, as these findings were not supported by consistent trends across related variables, their clinical and epidemiological relevance appears limited.

**Table 6:** Relationship between arch, type of supernumerary tooth and associated complications with supernumerary teeth.

Variable	Arch	Category	Frequency (*n*, %)	*p*-value
Type of supernumerary tooth	Maxilla	Mesiodens	8 (8.2%)	0.537
		Lateral incisors	7 (7.1%)	
		Canines	4 (4.1%)	
		Premolars	19 (19.4%)	
		Paramolars	2 (2%)	
		Distomolars	3 (3.1%)	
	Mandible	Mesiodens	7 (7.1%)	
		Lateral incisors	14 (14.3%)	
		Canines	11 (11.2%)	
		Premolars	19 (19.4%)	
		Paramolars	1 (1%)	
		Distomolars	3 (3.1%)	
Vertical location of cusp tip	Maxilla	Apical to root tip	0 (0%)	0.327
		Apical third	10 (10.2%)	
		Middle third	9 (9.2%)	
		Cervical third	18 (18.4%)	
		Coronal third	6 (6.1%)	
	Mandible	Apical to root tip	13 (13.3%)	
		Apical third	5 (5.1%)	
		Middle third	10 (10.2%)	
		Cervical third	12 (12.2%)	
		Coronal third	15 (15.3%)	
Buccolingual location of crown	Maxilla	Labial/Buccal	6 (6.1%)	0.031
		Median/within	18 (18.4%)	
		Palatal/Lingual	19 (19.4%)	
	Mandible	Labial/Buccal	2 (2%)	
		Median/within	20 (20.4%)	
		Palatal/Lingual	31 (31.6%)	
Shape of supernumerary tooth	Maxilla	Conical	7 (7.1%)	0.607
		Tuberculate	9 (9.2%)	
		Supplemental	22 (22.4%)	
		Odontoma	0 (0%)	
		Developing tooth bud	5 (5.1%)	
	Mandible	Conical	13 (13.3%)	
		Tuberculate	7 (7.1%)	
		Supplemental	28 (28.6%)	
		Odontoma	4 (4.1%)	
		Developing tooth bud	3 (3.1%)	
Position of supernumerary tooth	Maxilla	Normal	19 (19.4%)	0.001
		Inclined	18 (18.4%)	
		Transverse	6 (6.1%)	
		Inverted	0 (0%)	
		Undefinable	0 (0%)	
	Mandible	Normal	15 (15.3%)	
		Inclined	19 (19.4%)	
		Transverse	10 (10.2%)	
		Inverted	3 (3.1%)	
		Undefinable	8 (8.2%)	
State of eruption	Maxilla	Erupted	15 (15.3%)	0.099
		Impacted	28 (28.6%)	
	Mandible	Erupted	11 (11.2%)	
		Impacted	44 (44.9%)	
Degree of root resorption	Maxilla	No resorption	39 (14.7%)	0.465
		Slight	0 (0%)	
		Moderate	4 (1.5%)	
		Severe	0 (0%)	
	Mandible	No resorption	47 (48%)	
		Slight	3 (3.1%)	
		Moderate	2 (2%)	
		Severe	3 (3.1%)	
Root resorption of adjacent teeth	Maxilla	No resorption	39 (39.8%)	0.184
		Cervical	0 (0%)	
		Middle	4 (4.1%)	
		Apical	0 (0%)	
	Mandible	No resorption	45 (45.9%)	
		Cervical	2 (2%)	
		Middle	4 (4.1%)	
		Apical	4 (4.1%)	
Associated complications	Maxilla	Asymptomatic	6 (6.1%)	0.138
		Spacing	5 (5.1%)	
		Crowding	8 (8.2%)	
		Malposition	8 (8.2%)	
		Diastema	1 (1%)	
		Impaction	7 (7.1%)	
		Delayed eruption	7 (7.1%)	
		Cyst	1 (1%)	
		Other	0 (0%)	
	Mandible	Asymptomatic	6 (6.1%)	
		Spacing	8 (8.2%)	
		Crowding	4 (4.1%)	
		Malposition	6 (6.1%)	
		Diastema	0 (0%)	
		Impaction	15 (15.3%)	
		Delayed eruption	15 (15.3%)	
		Cyst	0 (0%)	
		Other	1 (1%)	

## Discussion

ST are an important dental condition that can affect normal tooth eruption and occlusion (2, 6). CBCT provides better detection and evaluation of ST compared with conventional imaging methods (15, 29). The principal findings of this study suggest that CBCT can identify more cases and give more detailed information about ST in this population. The main finding is that the prevalence of ST was high (36.6%), with most cases found in the mandibular premolar region and commonly associated with impaction and eruption-related complications.

The prevalence of ST is highly dependent on diagnostic methods and population characteristics. Traditional panoramic radiographs report prevalence rates of 0.3%–2.5% (29), whereas CBCT-based studies generally report higher detection rates due to superior imaging capabilities. For example, He et al. reported 6.67% prevalence in 13,336 Chinese children, whereas Zhao et al. found 1.24% among 48,700 outpatients using panoramic imaging (30, 31). CBCT studies such as Lykousis et al. observed ST in 11.6% of 224 patients aged 8–18 years (32). In our study, ST were detected in 36.6% of the population, markedly exceeding the global prevalence range. This elevated rate likely reflects both the enhanced sensitivity of CBCT in detecting non-erupted or atypically positioned teeth and potential selection bias inherent to CBCT datasets, which are often obtained for patients with complex dental needs rather than a random population sample.

Therefore, our findings may overestimate true population prevalence and should be interpreted with caution. Additionally, diagnostic imaging indications—such as assessment of impacted teeth or orthodontic planning—may have further influenced prevalence estimates, underscoring the importance of considering selection factors when comparing with other studies.

Interestingly, females (54.1%) slightly outnumbered males (45.9%) in this sample, contrasting with most reports indicating male predominance with ratios up to 2:1 (33). This discrepancy may reflect population-specific genetic and environmental influences, sampling differences, or variability in referral patterns for CBCT imaging. Similarly, the mandibular arch was more frequently affected (19.0%) than the maxilla (16.2%), contrary to previous studies emphasizing maxillary predilection, especially for anterior ST types like mesiodens (2, 34). The higher detection of posterior premolars in our CBCT sample may partly explain this pattern, highlighting how imaging modality and sampling strategy can shape observed distribution. A modest left-side predilection (20.5% vs. 16.0%) was also noted, consistent with some prior reports, although the clinical relevance of laterality asymmetry remains uncertain (1).

Premolars were the most prevalent ST type (38.8%), followed by lateral incisors (21.4%), mesiodens (15.3%), and canines (15.3%), diverging from traditional findings where mesiodens predominate (10, 35). Supplemental morphology (51.0%) dominated, followed by conical (20.4%) and tuberculate forms (16.3%), which aligns with previous CBCT studies emphasizing detection of morphologically complete teeth resembling normal dentition (36). Odontomas (4.1%) and developing tooth buds (8.2%) were also identified, likely reflecting the superior sensitivity of CBCT in detecting early or incomplete formations that might be missed on standard radiographs (1, 37–40).

The spatial distribution of ST also illustrates the utility of 3D imaging. Vertically, the cervical third of the root was most frequently involved (30.6%), followed by coronal (21.4%) and middle third (19.4%) positions. Buccolingually, ST were predominantly palatal/lingual (51.0%), consistent with prior CBCT reports (7). Orientation analysis showed a predominance of inclined (37.8%) and normally positioned (34.7%) teeth, with transverse and inverted forms less common but clinically significant due to surgical challenges (41). Impaction was highly prevalent (73.5%), particularly in males and on the right side, mirroring previous findings (42, 43). These observations reinforce that CBCT provides a comprehensive view of ST morphology and position, which is crucial for anticipating clinical complications.

Complications in this cohort included delayed eruption and impaction of adjacent teeth (17.3% each), with gender differences noted: males had more impaction, while females exhibited more crowding and malposition (*p* < 0.001). Root resorption, although relatively rare, was more prevalent in females (*p* = 0.022), consistent with prior CBCT-based studies (37). Regression analysis revealed no significant predictive effect of gender on the presence of anomalies (*p* = 0.306), supporting the multifactorial nature of ST development.

Overall, while several statistically significant associations were identified in subgroup analyses, most findings were inconsistent and exploratory in nature. The results therefore primarily highlight descriptive patterns rather than strong epidemiological relationships, underscoring the need for more focused, hypothesis-driven analyses in future studies.

## Clinical implications

This study highlights regional variations in ST among the Saudi population, with a higher prevalence in the mandibular arch and posterior regions. The elevated impaction rate (36.6%) and frequent eruption disturbances emphasize the need for early detection, with CBCT proving significantly more effective than traditional radiographs. CBCT enables accurate 3D localization of complex or unerupted ST, improving diagnostic precision, treatment planning, and surgical safety. Gender and arch-specific trends were observed—males had more impactions and cervical-positioned teeth, while females showed more crowding and root resorption. The predominance of mandibular premolars contrasts with earlier findings favoring maxillary mesiodens. Morphologically complex ST require careful evaluation to prevent surgical complications and adjacent tooth damage. A multidisciplinary, individualized approach is essential for optimal management and patient outcomes.

Although this study provides comprehensive epidemiological insights into ST using CBCT, several methodological limitations should be considered when interpreting the findings. Firstly, as the study was based on retrospective CBCT data and included only patients with specific clinical indications rather than a general population sample, the relatively high number of cases may be influenced by selection and referral bias. Secondly, the inclusion of a limited number of centers from the Ha’il region may restrict the generalizability of the findings to other populations. Thirdly, the lack of detailed clinical data limited the ability to correlate radiographic findings with patient symptoms and clinical outcomes. Fourthly, the regression analysis was restricted, as only gender could be included in the logistic model, while variables such as arch and side were not analysed due to their interdependence within patients and insufficient subgroup sizes. Finally, the absence of longitudinal follow-up prevented assessment of long-term outcomes and lesion progression.

## Conclusions

CBCT showed a high prevalence (36.6%) of supernumerary teeth in this Saudi sample, mainly in the mandibular premolar region, with variations by gender and dental arch. Females exhibited a greater incidence of root resorption and crowding, whereas males displayed increased impaction; however, these results necessitate careful interpretation. CBCT remains valuable for detailed evaluation and treatment planning.

## Data Availability

The raw data supporting the conclusions of this article will be made available by the authors, without undue reservation.

## References

[1] Mossaz J Kloukos D Pandis N Suter VG Katsaros C Bornstein MM. Morphologic characteristics, location, and associated complications of maxillary and mandibular supernumerary teeth as evaluated using cone beam computed tomography. Eur J Orthod. (2014) 36(6):708–18. 10.1093/ejo/cjt101 24385409

[2] Garvey MT Barry HJ Blake M. Supernumerary teeth—an overview of classification, diagnosis and management. J Can Dent Assoc. (1999) 65(11):612–6. 10658390

[3] Ata-Ali F Ata-Ali J Peñarrocha-Oltra D Peñarrocha-Diago M. Prevalence, etiology, diagnosis, treatment and complications of supernumerary teeth. J Clin Exp Dent. (2014) 6(4):e414–8. 10.4317/jced.51499 25593666 PMC4282911

[4] Rajab LD Hamdan MA. Supernumerary teeth: review of the literature and a survey of 152 cases. Int J Paediatr Dent. (2002) 12(4):244–54. 10.1046/j.1365-263X.2002.00366.x 12121534

[5] Fernández Montenegro P Valmaseda Castellón E Berini Aytés L Gay Escoda C. Retrospective study of 145 supernumerary teeth. Med Oral Patol Oral Cir Bucal. (2006) 11(4):339–44. 16816819

[6] Brook AH. A unifying aetiological explanation for anomalies of human tooth number and size. Arch Oral Biol. (1984) 29(5):373–8. 10.1016/0003-9969(84)90163-8 6611147

[7] Anthonappa RP King NM Rabie AB. Aetiology of supernumerary teeth: a literature review. Eur Arch Paediatr Dent. (2013) 14(5):279–88. 10.1007/s40368-013-0082-z 24068489

[8] Bäckman B Wahlin YB. Variations in number and morphology of permanent teeth in 7-year-old Swedish children. Int J Paediatr Dent. (2001) 11(1):11–7. 10.1046/j.1365-263x.2001.00205.x 11309867

[9] Chen YH Cheng NC Wang YB Yang CY. Prevalence of congenital dental anomalies in the primary dentition in Taiwan. Pediatr Dent. (2010) 32(7):525–9. 21462766

[10] Fardi A Kondylidou-Sidira A Bachour Z Parisis N Tsirlis A. Incidence of impacted and supernumerary teeth-a radiographic study in a North Greek population. Med Oral Patol Oral Cir Bucal. (2011) 16(1):e56–61. 10.4317/medoral.16.e56 20711166

[11] Burhan AS Nawaya FR Katbi ME Al-Jawabra AS. Prevalence of supernumerary teeth in a nonsyndromic Syrian sample. J Egypt Public Health Assoc. (2015) 90(4):146–9. 10.1097/01.EPX.0000475614.20865.db 26854894

[12] Salem G. Prevalence of selected dental anomalies in Saudi children from Gizan region. Community Dent Oral Epidemiol. (1989) 17(3):162–3. 10.1111/j.1600-0528.1989.tb00014.x 2786794

[13] Ghaznawi HI Daas H Salako NO. A clinical and radiographic survey of selected dental anomalies and conditions in a Saudi Arabian population. Saudi Dent J. (1999) 11(1):8–13.

[14] Afify AR Zawawi KH. The prevalence of dental anomalies in the Western region of Saudi Arabia. ISRN Dent. (2012) 2012:837270. 10.5402/2012/837270 22778974 PMC3388344

[15] Liu DG Zhang WL Zhang ZY Wu YT Ma XC. Three-dimensional evaluations of supernumerary teeth using cone-beam computed tomography for 487 cases. Oral Surg Oral Med Oral Pathol Oral Radiol Endod. (2007) 103(3):403–11. 10.1016/j.tripleo.2006.03.026 17321454

[16] Järvinen S Lehtinen L. Supernumerary and congenitally missing primary teeth in Finnish children: an epidemiologic study. Acta Odontol Scand. (1981) 39(2):83–6. 10.3109/00016358109162264 6948487

[17] Tsai SJ King NM. A catalogue of anomalies and traits of the permanent dentition of southern Chinese. J Clin Pediatr Dent. (1998) 22(3):185–94. 9641090

[18] Niswander JD Sujaku C. Congenital anomalies of teeth in Japanese children. Am J Phys Anthropol. (1963) 21(4):569–74. 10.1002/ajpa.1330210413 14185534

[19] Al-Jabaa AH Aldrees AM. Prevalence of dental anomalies in Saudi orthodontic patients. J Contemp Dent Pract. (2013) 14(4):724–30. 10.5005/jp-journals-10024-1391 24309355

[20] Vani NV Saleh SM Tubaigy FM Idris AM. Prevalence of developmental dental anomalies among adult population of Jazan, Saudi Arabia. Saudi J Dent Res. (2016) 7(1):29–33. 10.1016/j.sjdr.2015.03.003

[21] Yassin SM. Prevalence and distribution of selected dental anomalies among Saudi children in Abha, Saudi Arabia. J Clin Exp Dent. (2016) 8(5):e485–90. 10.4317/jced.52870 27957258 PMC5149079

[22] AlHumaid J Buholayka M Thapasum A Alhareky M Abdelsalam M Bughsan A. Investigating prevalence of dental anomalies in Eastern Province of Saudi Arabia through digital orthopantomogram. Saudi J Biol Sci. (2021) 28(5):2900–6. 10.1016/j.sjbs.2021.02.023 34025167 PMC8117041

[23] Renugalakshmi A Vinothkumar TS Bokhari AM Almahdi S Almalki A Balla SB Prevalence of dental anomalies and its role in sex estimation among children of Jazan Region, Saudi Arabia. Children. (2023) 10(4):759. 10.3390/children10040759 37190008 PMC10136972

[24] Mallineni SK Aldhuwayhi S Deeban Y Almutairi KS Alhabrdi SN Almidaj MA Prevalence, occurrence, and characteristics of supernumerary teeth among the Saudi Arabian population using panoramic radiographs. Diagnostics. (2024) 14(22):2542. 10.3390/diagnostics14222542 39594208 PMC11592778

[25] Aljuaid TS Manjunatha BS Amith HV Alshehri RA Alharthi FB Kariri AM. Prevalence and distribution of selected developmental dental anomalies in Taif, Saudi population. J Public Health Res. (2021) 11(1):2132. 10.4081/jphr.2021.2132 34558880 PMC8874842

[26] de Oliveira Gomes CA Drummond SN Jham BC Abdo EN Mesquita RA. A survey of 460 supernumerary teeth in Brazilian children and adolescents. Int J Paediatr Dent. (2008) 18(2):98–106. 10.1111/j.1365-263X.2007.00862.x 18237292

[27] Luten JR. The prevalence of supernumerary teeth in primary and mixed dentitions. J Dent Child. (1967) 34:346–53. 5341572

[28] Song JX Wassell JT. Sample size for K 2×2 tables in equivalence studies using Cochran’s statistic. Control Clin Trials. (2003) 24(4):378–89. 10.1016/S0197-2456(03)00026-6 12865033

[29] Mallineni SK Anthonappa RP Jayaraman J King NM. Radiographic localization of supernumerary teeth: a narrative review. Front Dent Med. (2025) 6:1495025. 10.3389/fdmed.2025.1495025 40008254 PMC11847821

[30] He L Que G Yang X Yan S Luo S. Prevalence, clinical characteristics, and 3-dimensional radiographic analysis of supernumerary teeth in Guangzhou, China: a retrospective study. BMC Oral Health. (2023) 23(1):351. 10.1186/s12903-023-03032-9 37268939 PMC10239132

[31] Zhao L Liu S Zhang R Yang R Zhang K Xie X. Analysis of the distribution of supernumerary teeth and the characteristics of mesiodens in Bengbu, China: a retrospective study. Oral Radiol. (2021) 37(2):218–23. 10.1007/s11282-020-00432-3 32198663

[32] Lykousis A Pouliezou I Christoloukas N Rontogianni A Mitsea A Angelopoulos C. Supernumerary teeth in the anterior maxilla of non-syndromic children and adolescents: a retrospective study based on cone-beam computed tomography scans. Pediatr Rep. (2025) 17(3):52. 10.3390/pediatric17030052 40407577 PMC12101261

[33] Brook AH. Dental anomalies of number, form and size: their prevalence in British schoolchildren. J Int Assoc Dent Child. (1974) 5:37–53. 4535299

[34] Leco Berrocal MI Martín Morales JF Martínez González JM. An observational study of the frequency of supernumerary teeth in a population of 2000 patients. Med Oral Patol Oral Cir Bucal. (2007) 12(2):E134–8. 17322802

[35] Liu JF. Characteristics of premaxillary supernumerary teeth: a survey of 112 cases. ASDC J Dent Child. (1995) 62(4):262–5. 7593884

[36] Gurung D Sun JH Xie NN Sun TZ Shrestha M. Supernumerary teeth and their complications: a cone beam computed tomography study. JNDA. (2021) 21:46–51.

[37] Solares R. The complications of late diagnosis of anterior supernumerary teeth: case report. ASDC J Dent Child. (1990) 57(3):209–11. 2345215

[38] Goswami S. Prevalence and characteristics of supernumerary teeth in pediatric patients: a retrospective study. J Prim Care Dent Oral Health. (2023) 4(3):99–102. 10.4103/jpcdoh.jpcdoh_28_23

[39] Gürler G Delilbaşı C Delilbaşı E. Investigation of impacted supernumerary teeth: a cone beam computed tomography study. J Istanbul Univ Fac Dent. (2017) 51(3):18–24. 10.17096/jiufd.20098 PMC5624141 29114426

[40] Guo J Jumatai S Dai Y Sun J Gong Z. A cone-beam computed tomography study of supernumerary teeth. Digit Med. (2023) 9(2):e00007. 10.1097/DM-2023-00007

[41] Högström A Andersson L. Complications related to surgical removal of anterior supernumerary teeth in children. ASDC J Dent Child. (1987) 54(5):341–3. 3478360

[42] Di Biase DD. Midline supernumeraries and eruption of the maxillary central incisor. Dent Pract Dent Rec. (1969) 20(1):35–40. 5258919

[43] Asaumi JI Shibata Y Yanagi Y Hisatomi M Matsuzaki H Konouchi H Radiographic examination of mesiodens and their associated complications. Dentomaxillofac Radiol. (2004) 33(2):125–7. 10.1259/dmfr/68039278 15314006

